# What Is Breast in the Bone?

**DOI:** 10.3390/ijms17101764

**Published:** 2016-10-22

**Authors:** Carrie S. Shemanko, Yingying Cong, Amanda Forsyth

**Affiliations:** Department of Biological Sciences and Arnie Charbonneau Cancer Institute, 2500 University Dr, NW, University of Calgary, Calgary, AB T2N 1N4, Canada; congyingying@hotmail.com (Y.C.); an.forsyth@gmail.com (A.F.)

**Keywords:** bone metastasis, prolactin, prolactin receptor, breast cancer, Sonic Hedgehog, osteoclast, osteolytic metastasis, osteoclastogenesis

## Abstract

The normal developmental program that prolactin generates in the mammary gland is usurped in the cancerous process and can be used out of its normal cellular context at a site of secondary metastasis. Prolactin is a pleiotropic peptide hormone and cytokine that is secreted from the pituitary gland, as well as from normal and cancerous breast cells. Experimental and epidemiologic data suggest that prolactin is associated with mammary gland development, and also the increased risk of breast tumors and metastatic disease in postmenopausal women. Breast cancer spreads to the bone in approximately 70% of cases with advanced breast cancer. Despite treatment, new bone metastases will still occur in 30%–50% of patients. Only 20% of patients with bone metastases survive five years after the diagnosis of bone metastasis. The breast cancer cells in the bone microenvironment release soluble factors that engage osteoclasts and/or osteoblasts and result in bone breakdown. The breakdown of the bone matrix, in turn, enhances the proliferation of the cancer cells, creating a vicious cycle. Recently, it was shown that prolactin accelerated the breast cancer cell-mediated osteoclast differentiation and bone breakdown by the regulation of breast cancer-secreted proteins. Interestingly, prolactin has the potential to affect multiple proteins that are involved in both breast development and likely bone metastasis, as well. Prolactin has normal bone homeostatic roles and, combined with the natural “recycling” of proteins in different tissues that can be used for breast development and function, or in bone function, increases the impact of prolactin signaling in breast cancer bone metastases. Thus, this review will focus on the role of prolactin in breast development, bone homeostasis and in breast cancer to bone metastases, covering the molecular aspects of the vicious cycle.

## 1. Introduction

“What is bred in the bone will not come out of the flesh” (based on the proverb, England (in Latin) circa 1290). Essentially this has been interpreted to mean that what is inherited, cannot be concealed. The normal developmental pathways contribute to both normal cell and tissue function, but when misregulated during cellular transformation and cancerous progression, these pathways can function inappropriately and with great harm. “What is breast in the bone” refers to the innate signal transduction pathways in the breast that can influence bone biology inappropriately when transformed breast cells migrate to the bone. In particular, this review refers to the roles of prolactin (PRL) signaling that contribute to normal mammary gland function, which are subverted in cancer to contribute to the disease process. We recently discovered that breast cancer cells that migrated to the bone carry the PRL-receptor (PRLR) and that PRL signaling to breast cancer cells advances osteolytic osteoclast differentiation via the production of a soluble factor that modulates the bone [1]. Interestingly, breast cancer cells secrete bone modulating factors most likely because they are produced normally in the breast for breast developmental processes: a number of these factors are PRL-regulated.

PRL and its receptor have essential roles in mammary gland development, particularly maintenance of luminal progenitors and the final functional differentiation of the breast with the production of milk-secretory alveolar cells [2,3,4]. The predominant pathway associated with this differentiation has been Janus kinase-2 (JAK2) [5,6] and signal transducer and activator of transcription-(STAT)-5a (STAT5a) [7,8,9].

The effects of PRL on breast cancer and metastasis are not fully elucidated, and reports appear conflicting due to our lack of understanding of PRL biology. High levels of local [10] or serum PRL [10,11] are tumorigenic in mice. Large prospective human studies identified an association of high PRL serum levels with breast cancer risk [12,13]. Elevated serum levels of PRL at pregnancy may explain in part the increase in breast cancer risk that occurs over the short-term post-pregnancy [14]. In breast cancer patients, high PRL is associated with an overall worse survival [15,16,17] and with an increase in the occurrence of breast cancer metastasis [18,19,20]. The PRLR has been reported to be part of a poor gene signature [21] and is associated with poor prognosis [21,22]. Therefore, there are reproducible associations, in humans, of increased breast cancer risk, progression, metastases and treatment resistance with increased PRL and the PRLR.

In contrast, in vitro data have demonstrated both invasive [23] and invasive suppressive properties [24,25] of PRL signaling, and a gene signature developed from xenografted T47D cells identified a gene signature associated with good prognosis [26]. Recent large human studies have demonstrated that PRL or PRL receptor expression or a gene signature of PRL, PRLR, JAK2 and STAT5a is associated with a good prognosis [27,28]. It is becoming apparent that the biology of PRL signaling is very complex [29] and that the microenvironment can play an important role in PRL pathway selection and response [30,31,32].

Contributing to PRL signaling complexity are two major sources of PRL, endocrine and autocrine/paracrine [33,34], the second of which helps understand the historical example of the poor response of patients treated with bromocriptine, which interferes with PRL secretion from the pituitary gland. PRL is very specific in its binding to the PRLR [35,36], although other related hormones will interact with the PRLR, such as growth hormone and placental lactogen. Constitutively-active PRLR variants have been identified [37], although not in association with breast cancer [38]. There are multiple PRLR isoforms that differ in their signaling capacity and function [29]. It is clear that PRL signaling is intricate at many molecular levels.

Approximately 70%–80% of advanced breast cancer patients will experience a metastasis to the bone [39,40], and this severely impacts their quality of life and can lead to death. It represents an essential area of study that will also impact our understanding of latent and active metastases.

## 2. Breast Cancer Bone Metastases

### 2.1. Vicious Cycle

**Figure 1:**
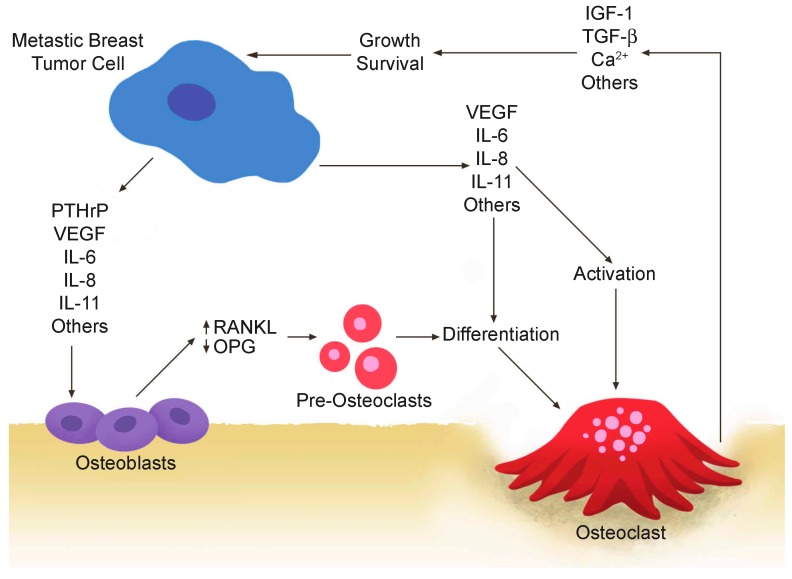
The vicious cycle of breast cancer bone metastases. Breast cancer cells secrete soluble factors, including parathyroid hormone-related protein (PTHrP), vascular endothelial growth factor (VEGF), interleukin (IL)-6, IL-8 and IL-11, which in the bone metastatic site act on osteoblasts and/or osteoclasts. The production of receptor activator of nuclear factor-κB ligand (RANKL), an osteoclast differentiation factor, is increased and the production of osteoprotegerin (OPG) is decreased from osteoblasts. Late-stage pre-osteoclast cells respond to specific breast cancer-secreted factors by differentiation and osteolytic activation.

The majority of breast cancer patients with bone metastases exhibit primarily osteolytic (bone destructive) lesions (80%–90%), although a small percentage (10%–20%) of patients exhibit primarily osteoblastic (bone building) lesions [41]. Breast cancer cells secrete soluble factors that act on osteoblasts or osteoclasts to ultimately stimulate osteoclast cell differentiation in lytic lesions. The osteoclasts then degrade the bone, which releases stored growth factors that stimulate breast cancer cell proliferation. The mitogenic factors, such as IGF-1, TGF-β and calcium, are stored during bone formation, and their action on the tumor cells perpetuates a vicious osteolytic cycle of tumor cell growth and osteolysis (Figure 1) [42]. The weakening of bone at the sites of metastasis puts the patients at risk of severe bone pain, bone breakage, spinal compression and hypercalcemia that result in pain and death [43]. The signaling pathways that feed into the vicious cycle have remained to be completely identified. Recently, a new PRL-based mechanism by which PRL-treated breast cancer cell-secreted factors directly promote the differentiation of functional osteoclast cells capable of bone resorption was identified, which likely is responsible for the observed acceleration of clinical detection of bone metastasis in patients with high levels of the PRLR [1]. Understanding the mechanism that accelerates the vicious cycle is key to therapeutic development.

Osteoclasts undergo both differentiation and functional activation as distinct stages and can morphologically differentiate without the ability to degrade bone matrix. Osteoclasts adhere to and migrate on the bone surface, synthesize and secrete hydrolytic enzymes, acidify the bone and internalize some degradation products [44]. To accomplish this, the osteoclast undergoes fusion to form multinucleate cells, cytoskeletal reorganization (actin ring) and polarization to establish a resorptive organelle called the ruffled border, which contacts the bone surface and constitutes the apical membrane. The cell then releases acidic components and lytic enzymes, such as tartrate-resistant acid phosphatase (TRAP). Many molecules act at different key steps in the process, such as macrophage colony stimulating factor (M-CSF), macrophage inflammatory protein-3 α (MIP3α), cardiotrophin-1 (CT-1) (for cell proliferation, survival, cell fusion, differentiation) [45,46,47] and receptor activator of nuclear-factor-κB (NFκB) ligand (RANKL) (for differentiation, survival and function), and other molecules are involved in attachment, actin cytoskeleton remodeling and the acidification process. The factors secreted by breast cancer cells in the presence of PRL stimulate osteoclast differentiation and lytic activity.

Three main pathways are essential for osteoclast differentiation, from the RANKL-RANK receptor, M-CSF-c-Fms and co-stimulatory signals [48,49]. Calcium and RANKL induce NFκB and the master regulator of osteoclastogenesis, nuclear factor of activated T cells-1 (NFATc1) [50], responsible for regulating the genes for TRAP, calcitonin receptor, cathepsin K and β3 integrin [49]. M-CSF upregulates the transcription factor c-fos via ERK [49]. Non-canonical pathways exist in arthritis, whereby RANKL can be substituted by “homologous to **l**ymphotoxins exhibiting inducible expression and competing with herpes simplex virus glycoprotein D for herpesvirus entry mediator (HVEM), a receptor expressed by T lymphocytes” (LIGHT), tumor necrosis factor α (TNFα), IL-6, IL-11 and IL-8 [51]. Therefore, it is possible that non-RANKL-dependent pathways exist.

Bone metastases is incurable and has limited treatment options. Current treatments include bisphosphonates. Despite treatment, over 50% of patients treated with bisphosphonates will have a recurrence with skeletal-related events [52]. The humanized monoclonal antibody against RANKL (Denosumab) offers improvement over certain bisphosphonates, reduces the risk of bone fracture in breast cancer patients by 50% and delays skeletal-related events [53]. Suboptimal therapy and ineffective anti-RANKL treatments in patient non-responders [54] may be explained by the presence of RANKL-independent pathways. Identification of a RANKL-independent pathway can offer the therapeutic option of co-targeting it and the RANKL pathway.

### 2.2. Osteoclastogenic Factors Produced by Breast Cancer Cells

Breast cancer cells secrete cytokines, such as parathyroid hormone-related protein (PTHrP) [55], vascular endothelial growth factor (VEGF) [56], interleukin (IL)-6 [57], IL-8 [58] and IL-11 [59] (Figure 1), which are known bone modulators. Breast cancer cells also secrete many others into the metastatic niche, which influence bone metastasis (Table 1). PTHrP is classically a major player, with elevated expression in tumors that have metastasized to the bone as opposed to tumors that have a visceral metastasis [60], although conflicting reports indicate that it is associated with non-bone metastasis and a good prognosis [61]. PTHrP interacts with receptors on osteoblasts that results in an increase in RANKL and decrease in osteoprotegerin (OPG) secretion. As OPG is an inhibitor of RANKL, the increase in the RANKL-OPG ratio favors osteolytic lesions [42]. RANKL is secreted by breast cancer cells in response to progesterone [62,63], although this mechanism has not been directly implicated in osteolysis. VEGF expression is present at high levels in breast cancer metastases to the bone, where it induces mature osteolytic osteoclasts [56,64] and increases their survival [65]. Il-6, IL-8 and IL-11 stimulate osteoclasts directly in addition to osteoblasts and bone stroma at the site of metastasis [66,67,68]. It is clear now that there are multiple breast cancer-secreted factors that can influence the osteolytic metastatic niche (Table 1), in general by usurping the natural production of factors that have multiple cell type-dependent roles.

### 2.3. Osteoblastic Factors Produced by Breast Cancer Cells

Although breast cancer is primarily osteolytic, the metastatic lesion can also result in a build-up of bone, which is also harmful. In these cases, breast cancer cells likely secrete factors that influence osteoblast function that results in bone build-up as opposed to osteoclast differentiation and bone loss. There are a number of potential factors that are not well understood in breast cancer, which are however better understood in prostate cancer, which is primarily osteoblastic [92]. At least one breast cancer-secreted factor, platelet-derived growth factor (PDGF)-β polypeptide B (BB), has been observed to be osteosclerotic, promoting bone density [93].

## 3. Prolactin in Bone Homeostasis: Pregnancy and Lactation

There are two main phases of bone changes associated with reproduction, with an increase in bone in early pregnancy and bone loss in later pregnancy and lactation in the mother in order to ultimately provide calcium to the milk. PRL appears to have a role in the late pregnancy and lactation-associated bone loss within the mother [94]. PRL may be responsible for bone loss at lactation [94,95,96], consistent with evidence [97,98,99] that demonstrates a direct effect of PRL on osteoblasts, which express the PRLR. These results are consistent with the phenotype of hyperprolactinemia patients, who experience bone loss [100], thought to be either due to the indirect effect of PRL that results in hypogonadism, and therefore, low estrogen, or alternatively by the direct action of PRL on osteoblasts [99,101]. PRL-PRLR directly increases bone turnover by raising the RANKL/OPG ratio [99,102]. PRL induction of osteoblasts was shown to increase the expression of RANKL, monocyte chemoattractant protein-1 (MCP-1), cyclooxygenase-2 (COX-2), ephrin-B1 (EPHB1), TNFα and IL-1 [101]. Interestingly, in fetal bone cells, PRL appears to promote bone gain in both rat and in human cells [97], resulting in an age-dependent response. In young rats, PRL was also shown to promote calcium absorption [103]. Osteoclasts do not express the PRLR [102], so any effect of PRL on osteoclasts would be indirect, including via PRLR+ osteoblasts or PRLR+ breast cancer cells. PRL can stimulate intestinal calcium and absorption and decreases renal calcium excretion [104]. Overall, PRL is considered to be a major calciotropic hormone that controls calcium mobilization from the bone and absorption in the gut [105].

## 4. Prolactin and the Regulation of Bone Modulating Factors in the Breast

RANKL was initially implicated as a PRL-STAT5 target gene in the mouse mammary gland, since RANKL expression was decreased in PRLR null mouse models [106], and induced by PRL [107,108]. The mammary gland phenotype of RANKL deletion [107] also resembles that of PRL-PRLR deficiency [3,4], in that there is a failed alveolargenesis and reduced ductal branching. Its pathway became more clear when it was determined that RANKL could rescue the progesterone receptor (PR) knockout mammary phenotype of side branching and alveolargenesis, and RANKL is a mediator of PR-E74-like factor 5 (ETS domain transcription factor) (ELF5)-induced alveolargenesis [109,110]. Recent work indicates that RANKL impairs PRL-STAT5 signaling and that this inhibition must be lifted in order for the functional differentiation of the mammary gland to take place [111]. Therefore, RANKL has two roles at different stage of mammary gland development, one mediating progesterone functions to promote early alveolargenesis and the second to help time lactogenic differentiation.

PTHrP [112], VEGF [113] and M-CSF [114] are upregulated by PRL in the mammary gland (Table 2). PTHrP is essential for calcium transport in the milk [115]. VEGF makes an important contribution to mammary gland function, in particular lobulo-alveolar expansion and milk production [116]. M-CSF is important for the lactating phenotype in the mammary gland and is also present in the milk [117]. It is likely that these well-known bone-modulators in the breast are important for PRL function.

**Figure 2:**
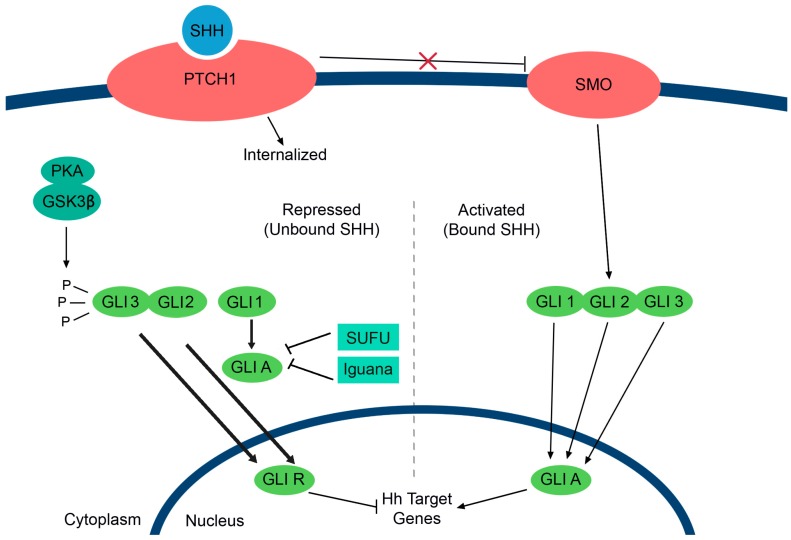
Hedgehog signaling. In the absence of Hedgehog (HH) ligand, the receptor Patched-1 (PTCH1) indirectly inhibits smoothened (SMO), resulting in the prevention of downstream signaling. Protein kinase-A (PKA) and glycogen synthase kinase β (GSK3β) phosphorylate GLI2/3 to help generate repressor GLI transcription factors (GLI R). Suppressor of fused (SUFU) and Iguana inhibit any activator GLI transcription factors (GLI A). Upon binding of the Sonic Hedgehog (SHH), Indian HH or Desert HH ligands, PTCH1 releases the inhibition of SMO (red X on the inhibitory line), and causes an accumulation of the activator GLI transcription factors and activation of HH target genes.

As discussed below, PRL also induces Sonic Hedgehog (SHH) at the protein level in breast cancer cells, although the signal transduction pathway is not known. SHH is produced in the breast although its function is not clearly linked to PRL. In fact, although there is SHH expression in the mammary gland, its function is not clear [118]. Hedgehog (HH) ligands, SHH, Indian HH and Desert HH, are expressed in the postnatal mammary gland. Components of the HH pathway (Figure 2) are required for ductal patterning and elongation [118]. Indian HH contributes to mammary epithelial expansion from stem and progenitor cells during pregnancy [119], consistent with PRL function. SHH is capable of activating non-canonical HH pathways in the mammary gland, such as extracellular regulated kinase (ERK) [120]. Hedgehog signaling, via cilia formation, is important in branching morphogenesis and pregnancy-induced alveolargenesis [121], consistent with indirect and direct PRL function. It will be interesting in the future to understand how these pathways interact.

## 5. The Role of Prolactin in Breast Cancer-Mediated Osteoclastogenesis

**Figure 3:**
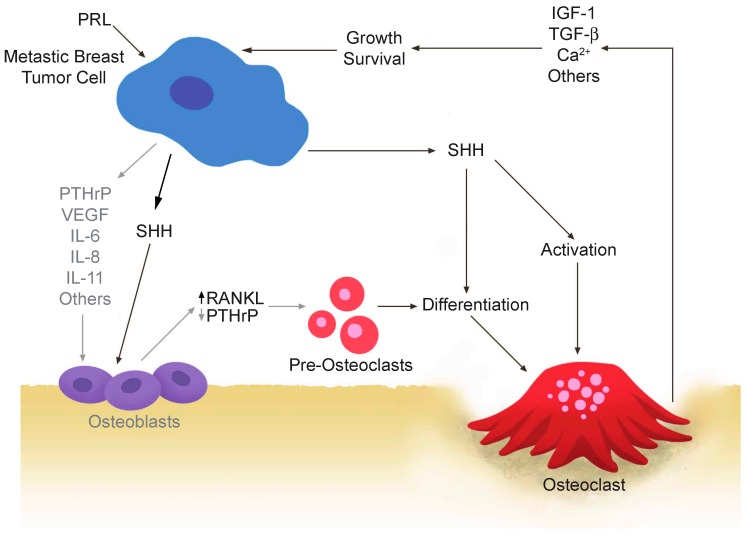
Prolactin (PRL)-stimulated breast cancer cells secrete SHH to induce the differentiation of lytic osteoclasts. High levels of the PRL-receptor (PRLR) in the primary tumor are associated with a shorter time to bone metastasis, presumably due to an acceleration of the vicious cycle.

We discovered that PRL can escalate the osteolytic aspect of the vicious cycle, by stimulating pre-osteoclasts indirectly to differentiate into osteolytic cells through breast cancer secreted factors, such as SHH [1] (Figure 3). High PRLR expression in the primary breast tumour was associated with a shorter time to the clinical presentation of bone metastasis. We also detected PRLR expression on circulating tumour cells in peripheral blood and in paired primary breast tumour and bone metastatic samples [1]. PRL and the PRLR enhanced the endogenous capacity of the breast cancer cells to directly induce the differentiation of osteoclasts via production of soluble factors and also contributed to their osteolytic ability. The molecular mechanism, in part, involved the PRL-induced upregulation of SHH at the protein level from breast cancer cells. Breast cancer-secreted SHH is capable of inducing osteoclast differentiation both directly and indirectly via osteoclasts or osteoblasts [88,89]. This is the first PRL-based molecular mechanism that has been established in breast cancer metastases, in particular to breast cancer metastases to the bone.

## 6. Future Directions and Impact: Therapeutic Implications

PRL signaling is complex, although it is clear that PRL could have an important impact on breast cancer bone metastases. The natural production of factors in the breast that also have roles in the bone is consistent with the cell type-specific control over a limited number of secreted factors in the body. Metastasis of cancer cells results in drastically altered microenvironments that affects multiple cell types and greatly alters normal homeostasis, resulting in an imbalance of osteolytic factors and the production of a lytic bone lesion. What is breast in the bone, in this case, is the PRL-regulated factors (and others) of transformed mammary epithelial cells that have metastasized to the bone, which usurp the normal homeostasis and push the local environment to bone loss. These observations also identify the PRLR as a potential therapeutic target in not only breast cancer, but for breast cancer bone metastases.

## Figures and Tables

**Table 1:** Breast cancer-secreted factors that induce differentiated osteoclasts indirectly or directly. Factors may act on osteoblasts to indirectly induce osteoclast differentiation or directly on osteoclasts.

Factor	Full Name	Target Cell	Reference
5HT	Serotonin	OB	[69]
ADAMTS1	A disintegrin and metalloproteinase with Thrombospondin motifs	OB	[70]
AREG	Amphiregulin	OB	[70]
CCL2/CCN2	Chemokine (C–C motif) ligand 2	stromal	[71]
CCN3	Cysteine-rich protein 61, connective tissue growth factor and nephroblastoma overexpressed	OB	[72]
CTGF	Connective tissue growth factor	–	[73]
DKK-1	Dickkopf-1	–	[74]
M-CSF	Macrophage-colony stimulating factor	OC	[75]
GM-CSF	Granulocyte macrophage-colony stimulating factor	OC	[76]
HB-EGF	Heparin-binding epidermal growth factor	OB	[70]
IGF-1	Insulin-like growth factor	–	[77]
IL-6	Interleukin-6	OB, OC	[57]
IL-8	Interleukin-8	OB, OC	[58,78]
IL-11	Interleukin-11	OB, OC	[79]
Jagged-1	Jagged-1	OB, OC	[80]
MMP-1	Matrix metalloproteinase-1	OC	[70,81]
MMP-9	Matrix metalloproteinase-9	–	[82]
OSM	Oncostatin-M	OC	[83]
–	Oxygen-derived free radical	OC	[84]
PDGF	Platelet-derived growth factor	–	[85]
PRDX4	Peroxiredoxin-4	OC	[86]
PTHrP	Parathyroid hormone-related protein	–	[55]
RANKL	Receptor activator of nuclear factor-κB ligand	OC	[62,63]
Sema4D	Semaforin-4D	OB	[87]
SHH	Sonic hedgehog	OB, OC	[88,89]
TGF-α	Transforming growth factor-α	OB	[70]
TGF-β	Transforming growth factor-β	–	[90]
VCAM1	Vascular cell adhesion molecule-1	OC	[91]
VEGF	Vascular endothelial growth factor	OC	[64]

OB, osteoblasts; OC, osteoclasts.

**Table 2:** PRL-regulated factors in the mammary gland, known to have a role in the bone.

Factor	Full Name
PTHrP	Parathyroid hormone-related protein
VEGF	Vascular endothelial growth factor
M-CSF	Macrophage colony stimulating factor
RANKL	receptor activator of nuclear factor-κB

## Abbreviations

CT-1	cardiotrophin
COX-2	cyclooxygenase-2
ELF5	E74-like factor 5 (ETS domain transcription factor)
EPHB1	ephrin-B1
ERK	extracellular regulated kinase
GSK3β	glycogen synthase kinase-3β
JAK2	Janus kinase-2
HH	hedgehog
IGF	insulin-like growth factor
IL	interleukin
LIGHT	homologous to lymphotoxins exhibiting inducible expression and competing with herpes simplex virus glycoprotein D for herpesvirus entry mediator (HVEM), a receptor expressed by T lymphocytes
MCP-1	monocyte chemoattractant protein-1
M-CSF	macrophage colony stimulating factor
MIP3α	macrophage inflammatory protein-3 α
NFATc1	nuclear factor of activated T cells-1
OPG	osteoprotegerin
PDGF-BB	platelet-derived growth factor β polypeptide B
PKA	protein kinase-A
PR	progesterone receptor
PRL	prolactin
PRLR	prolactin receptor
PTCH1	patched-1
PTHrP	parathyroid hormone-related protein
RANKL	receptor activator of nuclear factor-κB (NFκB) ligand
SMO	smoothened
STAT5a	signal transducer and activator of transcription-5a
SUFU	suppressor of fused
TGF-β	transforming growth factor-β
TNFα	tumor necrosis factor α
TRAP	tartrate-resistant acid phosphatase-1
VEGF	vascular endothelial growth factor
